# A Covalent Organic Framework With Staggered Topology for Mimetic Transmembrane Transport of Hydrated Ions

**DOI:** 10.1002/advs.76918

**Published:** 2026-08-05

**Authors:** Xuan Li, Jiaxin Liu, Yilin Zhang, Zhixiang Dai, Zihan Tan, Hongli Yang, Chao Xu, Shengyang Zhou, Zhong‐Ming Li

**Affiliations:** ^1^ College of Materials Science and Engineering College of Polymer Science and Engineering State Key Laboratory of Advanced Polymer Materials Sichuan University Chengdu China; ^2^ Department of Materials Science and Engineering The Ångström Laboratory Uppsala University Uppsala Sweden

**Keywords:** battery (electricity), covalent organic framework, electrolyte, ion transportation, membrane

## Abstract

In biological systems, hydrated‐ions cross protein channels with exceptional high efficiency through a structural dehydration‐compensation mechanism, where protein conformational adjustments contract the channel and enable electron‐rich sites to coordinate with ions, offsetting their dehydration energy without external energy input. In this work, we discovered that a typical *β*‐ketoenamine‐linked covalent organic framework (COF) with staggered‐stacking architectures exhibits a similar mechanism. Although COFs lack dynamic conformational adjustments, the AB‐staggered topology generates convergent channels enriched with carbonyl groups, which provide out‐sphere‐like multisite interactions with hydrated ions, partially compensating for the hydration dissociation energy and enabling rapid and efficient ion transport with low solvation. When employed as separators in aqueous zinc batteries, AB‐staggered COF membranes simultaneously suppress parasitic reactions and zinc dendrite formation. Zn anodes paired with these COF membranes exhibit markedly enhanced electrochemical cycling reversibility with stable Zn stripping/plating for over 5000 h in a conventional ZnSO_4_ electrolyte without any additives, outperforming most reported aqueous battery separators. This study identifies and experimentally validates rapid, low‐solvation hydrated‐ion transport in COFs via a biomimetic structural dehydration‐compensation mechanism, establishing a conceptual foundation for the rational design of high‐performance COF membranes in multiple fields, including but not limited to electrochemical energy storage, chemical separation, and catalytic systems.

## Graphical abstract

Inspired by biological ion channels, an AB‐staggered β‐ketoenamine‐linked COF enables rapid hydrated‐ion transport through a biomimetic dehydration‐compensation mechanism, where carbonyl‐rich convergent channels partially offset ion dehydration energy. As aqueous Zn battery separators, the COF membranes suppress parasitic reactions and dendrite growth, achieving highly reversible Zn stripping/plating over 5000 h without electrolyte additives.

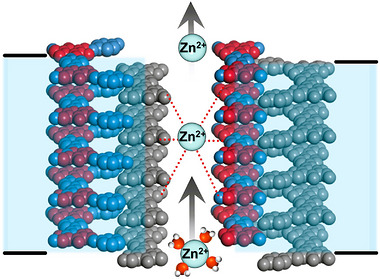

## 1 Introduction

**FIGURE 1:**
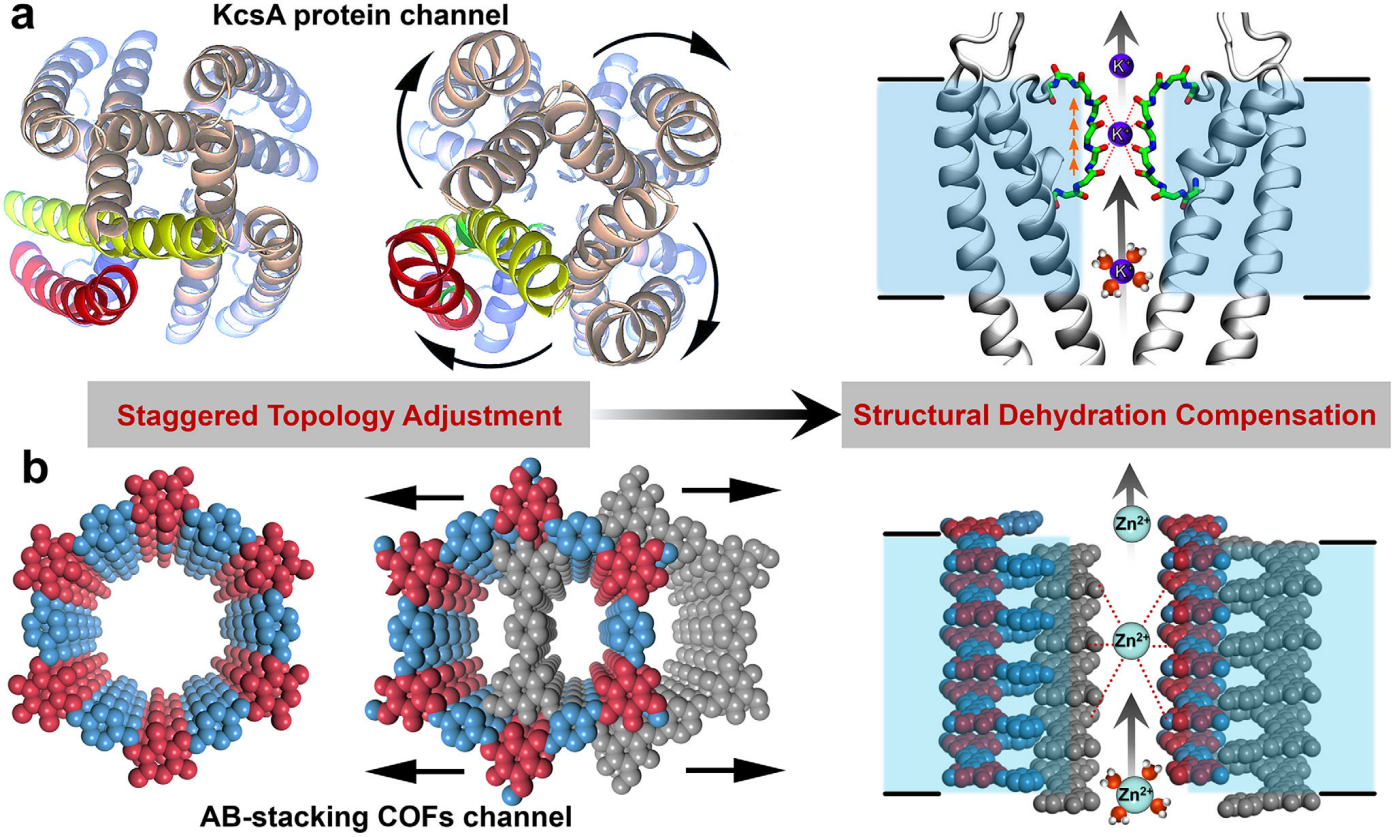
Schematic illustration of (a) passive K^+^ transmembrane transport in the KcsA protein channel via a typical dehydration‐compensation mechanism [14, 15], and (b) the analogous Zn^2+^ transport through *β*‐ketoenamine‐linked COF channels with a staggered conformation.

Ion channels within biological membranes precisely regulate the transmembrane transport of ions and water molecules with remarkable selectivity and efficiency, enabling low‐energy transport that maintains both transmembrane potential and osmotic balance [1, 2, 3]. Based on classical transition state theory (TST), this regulation relies on a unique structural dehydration compensation mechanism, in which electron‐rich groups lining the interior of the protein channel transiently coordinate with permeating ions (Figure 1a) [4, 5, 6]. The protein channel can adjust its conformation to achieve spatially optimized multisite coordination, allowing these electron‐rich groups to engage the ion in a controlled, multi‐dentate manner that perfectly compensates for the energy required to remove the hydration shell. The energy released from this coordinated binding fully offsets the desolvation penalty, enabling rapid, uniform, and energy‐efficient ion transport under aqueous conditions. In fact, such a behavior of ion transport corresponds to the optimal scenario desired for aqueous battery systems. As a representative example of the extensively studied aqueous zinc‐ion batteries in recent years, Zn^2+^ forms the stable [Zn(H_2_O)_6_]^2+^ complex in the commonly used 2 m ZnSO_4_ electrolyte, which imposes a high desolvation energy barrier of approximately 400 kJ mol^−1^ [7, 8, 9, 10]. The slow kinetics of desolvation during electrochemical deposition induce interfacial polarization and allow unremoved water molecules to participate in competing parasitic reactions, such as hydrogen evolution, which disrupts uniform zinc nucleation [11, 12, 13]. These coupled phenomena create a self‐amplifying cycle of dendrite growth and electrolyte depletion, ultimately compromising battery stability. The biological dehydration‐compensation strategy suggests that incorporating analogous passive ion transport pathways into aqueous batteries could address these challenges by lowering desolvation barriers while maintaining directional ion flux. Despite its promising potential to enhance aqueous battery performance, research exploring these bioinspired passive transport strategies remains scarce.

Constructing biomimetic transmembrane ion transport pathways in artificial materials remains highly challenging due to the difficulty of replicating the highly ordered, crystalline channels in protein membranes and the complexity of optimizing ion‐functional group interactions through precise control of pore topology. Achieving spatially arranged, electron‐rich chemical sites that can dynamically interact with ions and compensate for their dehydration energy requires structural precision that is rarely realized in synthetic materials. Herein, we discover that a typical *β*‐ketoenamine‐linked covalent organic framework (COF) with staggered stacking architectures can mimic key aspects of biological transmembrane passive ion transport (Figure 1b) [16, 17, 18, 19]. This AB‐stacking COF forms convergent channels enriched with carbonyl groups, which serve as electron‐rich sites for zinc ions. Although it cannot dynamically adjust the channel topology like biological pores to fully compensate for dehydration energy, the staggered topology enables compact coordination‐like multisite interactions that partially stabilize dehydrated ions in a manner similar to protein channels. These interactions offset dehydration energy and facilitate rapid, low‐energy, and uniform ion transport through the confined channels. This biomimetic mechanism effectively realizes the desolvation of zinc ions and homogenizes its flux at the electrode interface, which benefits the suppression of dendrite formation and minimizes parasitic reactions. Electrochemical evaluation reveals that AB‐stacked COF membranes enable ultra‐long cycle stability of zinc anodes, exceeding 5000 h in a conventional 2 m ZnSO_4_ electrolyte without any additives. In the full battery test by employing V_2_O_5_ as cathodes, the capacity retention remains above 90% after 1000 cycles at a large current density of 1 A g^−1^ with Coulombic efficiency consistently near 100%, significantly outperforming most of the recently reported separators. This study demonstrates a desolvation ion transport phenomenon in staggered COF membranes similar to transmembrane ion transport in biological systems and highlights the potential of topologically controlled COFs to overcome inherent desolvation and interfacial challenges in aqueous batteries. It provides a clear pathway for translating biomimetic principles into high‐performance, stable devices while maintaining fast and energy‐efficient ion transport.

## 2 Results and Discussion

### 2.1 Synthesis and Characterization of TPDB‐AA and TPDB‐AB Membranes

**FIGURE 2:**
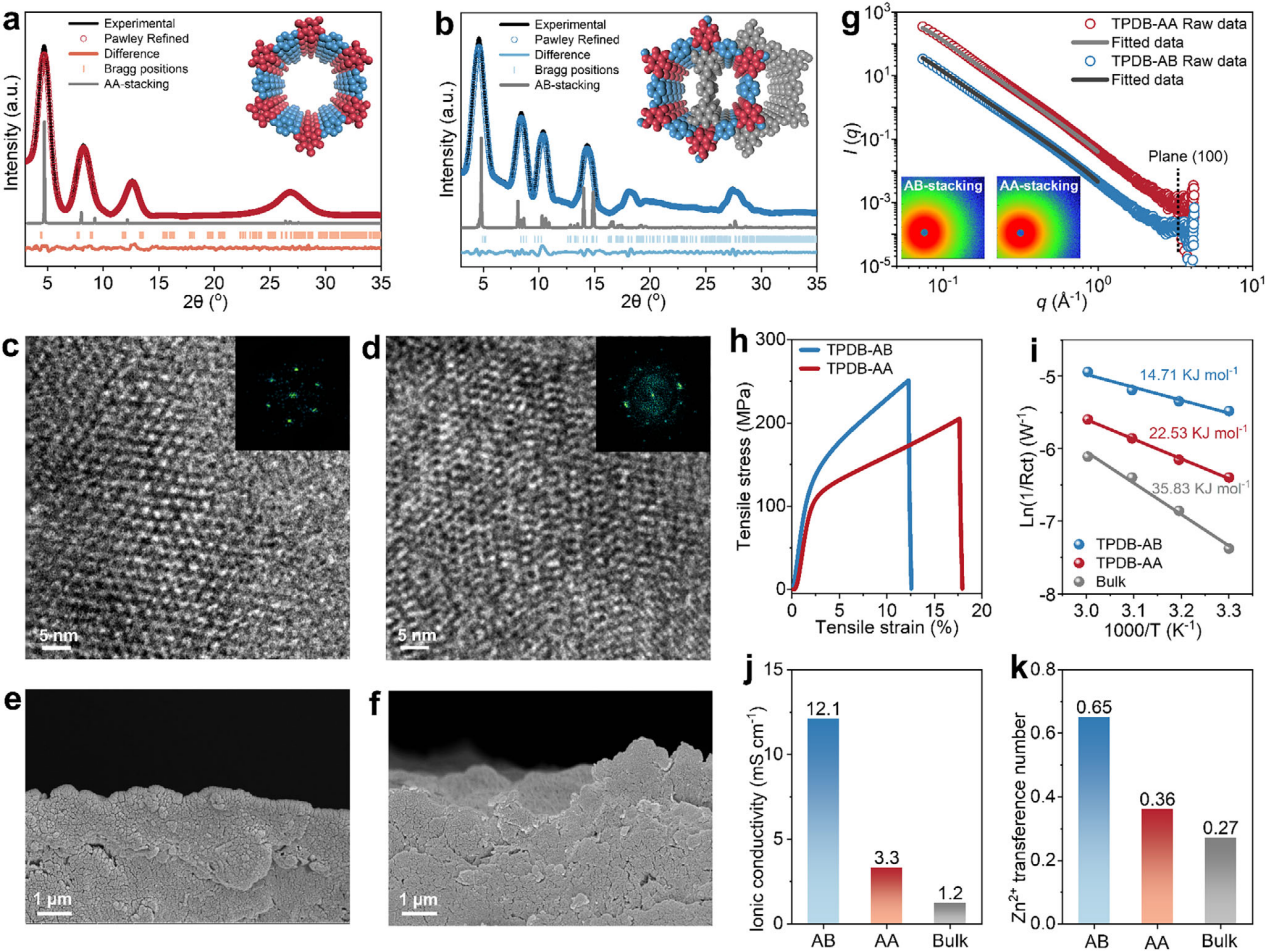
Structural and ion transport characterization of TPDB‐AA and TPDB‐AB COF membranes. (a,b) Experimental and simulated XRD patterns with corresponding molecular models. (c,d) HR‐TEM images of two COFs. (e,f) SEM images, (g) 2D SAXS patterns and 1D intensity curves, and (h) mechanical tensile stress‐strain curves of two fabricated COF membranes. (i) Arrhenius activation energies, (j) ionic conductivities, and (k) zinc ion transference numbers of two COF membranes compared with bulk electrolyte.

*β*‐Ketoenamine‐linked covalent organic frameworks (COF) with distinct interlayer stacking modes, denoted as TPDB‐AA and TPDB‐AB, were synthesized via a one‐step aqueous approach using 2,4,6‐triformylphloroglucinol (TP) and benzene‐1,4‐diamine (DB) as linkers. When 6 m acetic acid was used as the catalyst, the conventional AA‐stacked COF was obtained, whereas a mixed catalytic system consisting of 6 m acetic acid and 1 mg mL^−1^ polyoxometalates acid directed the formation of the AB‐stacked architecture, as illustrated in Figures S1 and S2. The crystallographic structures of the two COFs were first examined by X‐ray diffraction (XRD) (Figure 2a,b). TPDB‐AA exhibits well‐defined diffraction peaks at 4.52°, 8.44°, and 26.58°, corresponding to the (100), (110), and (001) planes, respectively [20, 21]. The sharp reflections indicate a high degree of crystallinity and match the simulated AA‐stacked model, confirming the typical AA stacking configuration. In contrast, TPDB‐AB displays a more complex diffraction pattern with reflections at 4.86°, 8.64°, 10.11°, 14.87°, and 27.44°. Compared to TPDB‐AA, peak shifts and additional features are evident. Comparison with simulated patterns shows excellent agreement with an AB‐stacked model, unambiguously identifying the staggered stacking topology. High‐resolution transmission electron microscopy (HR‐TEM) further confirms the distinct crystalline structures (Figure 2c,d). TPDB‐AA exhibits a honeycomb‐like lattice with long‐range order characteristic of a hexagonal crystalline framework, and the corresponding fast Fourier transform (FFT) pattern displays sharp, regularly arranged diffraction spots, consistent with AA stacking. TPDB‐AB presents a more intricate stacking lattice with pronounced lattice distortion and interlayer offset. COF layer maintains a hexagonal honeycomb framework, but staggered stacking between layers produces FFT patterns showing offset and partially crossed hexagonal diffraction spots, indicative of AB‐staggered topology and consistent with XRD simulations.

The synthesized COF powders could be fabricated into freestanding membranes via vacuum filtration with only a small amount of nanocellulose serving as a binder (Schematic S1, Figure S3). The preparation details are available in the Supporting Information and our previous works [22]. To ensure the reliability of the comparative study, both COF membranes were prepared using strictly identical procedures, so that the cellulose binder content, membrane thickness, and overall composition were kept the same (Figure S4). Scanning electron microscopy (SEM) images reveal that both COF membranes exhibit densely packed and uniform crystalline particle morphologies (Figure 2f,g). Energy‐dispersive X‐ray spectroscopy (EDS) analysis further confirms the uniform distribution of COF crystallites within the membranes (Figures S5 and S6). Small‐angle X‐ray scattering (SAXS) was further employed to finely analyze the pore structures of the two TPDB COF membranes (Figure 2c). Detailed fitting of the Guinier region indicates that TPDB‐AA exhibits a pore size of approximately 2 nm, whereas TPDB‐AB shows a significantly smaller pore size of around 1.4 nm, consistent with the XRD simulations and TEM observations. Nitrogen adsorption‐desorption measurements were additionally performed to validate the pore structures (Figures S7 and S8). The nitrogen adsorption analysis shows that TPDB‐AA membrane possesses a higher specific surface area with pore size centered near 2 nm, while the TPDB‐AB membranes exhibit a reduced surface area and pore size of near 1.5 nm, consistent basically with the characteristic dimensions derived from SAXS measurements. These results confirm that the interlayer stacking arrangement strongly influences the nanoporous architecture of the COF, and that the AB‐staggered topology leads to the formation of smaller and more compact pores.

Mechanical testing shows that TPDB‐AA and TPDB‐AB COF membranes exhibit tensile strengths exceeding 200 MPa and Young's moduli above 5 GPa, reflecting their robust mechanical integrity derived from the highly crystalline frameworks and dense interparticle packing (Figure 2h). Water contact angle measurements indicate that COF membranes with both stacking arrangements exhibit excellent hydrophilicity, facilitating efficient electrolyte wetting and ion transport (Figure S9). The hydrated ion transport properties of the two COF membranes were systematically evaluated using Zn^2^^+^ as a model ion in aqueous electrolyte. As shown in Figures 2i and S10, the Arrhenius activation energies for Zn^2+^ migration through the two COF membranes are significantly lower than in the bulk electrolyte, decreasing from 35.83 kJ mol^−1^ in the bulk to 22.53 kJ mol^−1^ for TPDB‐AA and 14.71 kJ mol^−1^ for TPDB‐AB. These results indicate that both COF membranes facilitate faster ion transport, with the TPDB‐AB membrane exhibiting the higher ion mobility. Consistently, the ionic conductivity of TPDB‐AB reaches 12.1 mS cm^−1^, markedly higher than that of TPDB‐AA (3.3 mS cm^−1^) and the bulk electrolyte (1.2 mS cm^−1^) (Figure 2j and Figure S11). In addition, the zinc ion transference numbers for TPDB‐AB and TPDB‐AA are measured to be 0.65 and 0.36, respectively, both exceeding the value of the bulk electrolyte (0.27) (Figure 2k and Figure S12). In addition, to exclude the influence of the cellulose binder and trace residual POMs on the ion transport, comparative experiments were also performed using pure cellulose and cellulose/POM (1 wt.%) composite membranes (Figures S13 and S14). Both membranes had minimal effects on Zn^2+^ conductivity, with transport behavior largely consistent with that of the bulk ZnSO_4_ electrolyte. The above results indicate that the enhanced Zn^2+^ conduction in the COF membrane mainly originates from the COF channels, rather than from the cellulose binder or the residual POMs.

### 2.2 Ion Transmembrane Transport Mechanisms

**FIGURE 3:**
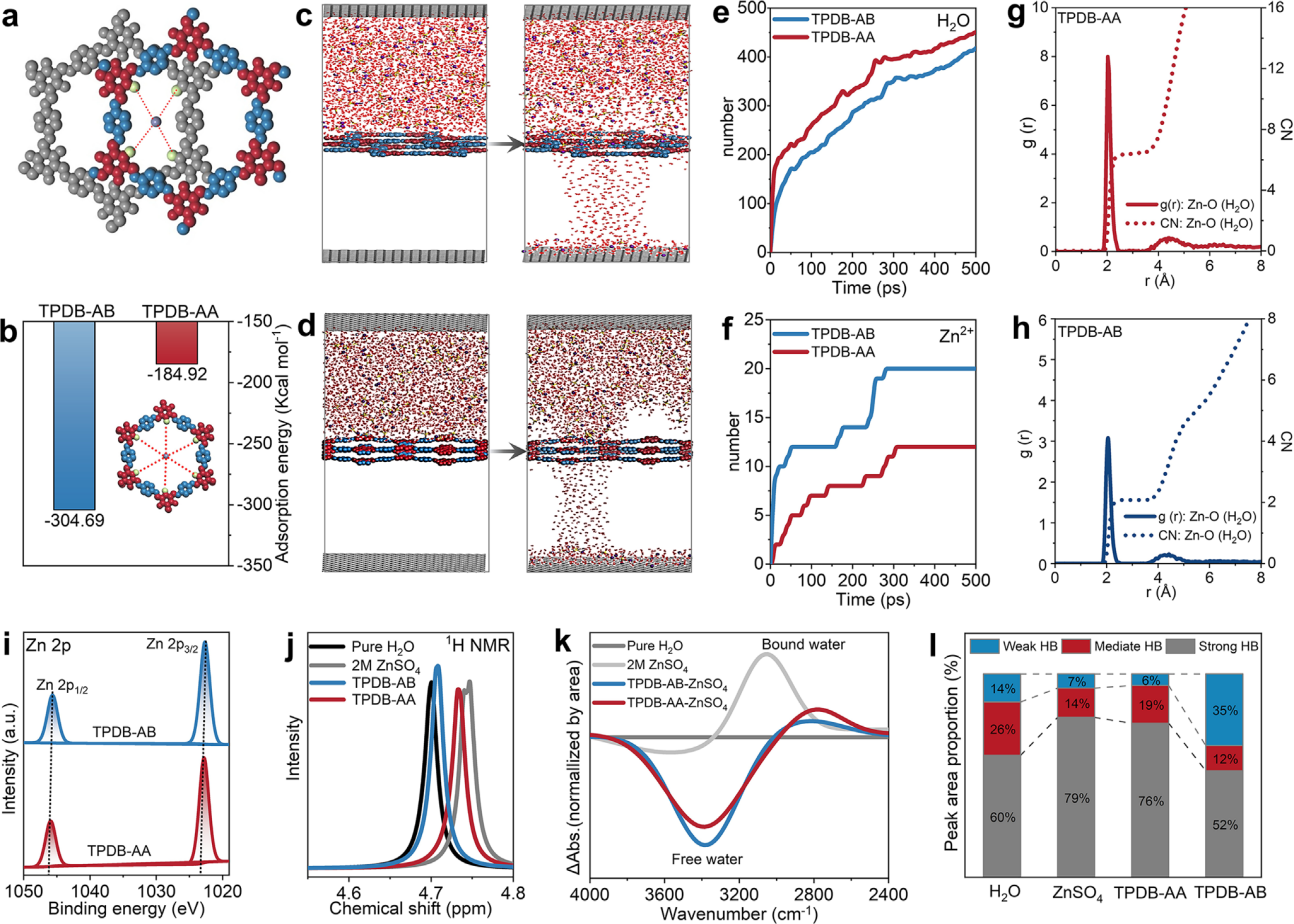
Mechanism of ion transmembrane transport through TPDB‐AA and TPDB‐AB membranes. (a) Molecular model used for density functional theory calculations of the adsorption energy between Zn^2+^ and the TPDB‐AB framework. (b) Calculated adsorption energies between Zn^2+^ and different COF. Molecular dynamics simulations of aqueous ZnSO_4_ electrolyte transport through the two COF membranes, including representative snapshots of 2 m ZnSO_4_ transport through (c) TPDB‐AB and (d) TPDB‐AA membranes. Time‐dependent transport numbers of (e) H_2_O molecules and (f) Zn^2+^ ions through the two COF membranes. Simulated radial distribution functions and coordination numbers of Zn^2+^ after passing through the (g) TPDB‐AA and (h) TPDB‐AB membranes. (i) Zn 2p XPS spectra of dried ZnSO_4_ confined within the COF membranes. (j) ^1^H NMR spectra, (k) ATR‐FTIR spectra, and (l) calculated proportions of hydrogen bonds in pristine water, bulk ZnSO_4_ electrolyte, and the two COF membranes.

To understand the Zn^2+^ transport mechanism within COF membranes with distinct stacking topologies, density functional theory (DFT) calculations were conducted to quantify the interaction energies between Zn^2+^ and carbonyl groups in TPDB frameworks (Table S1). As illustrated in Figure 3a, the bilayer AB‐stacked COF enables Zn^2+^ to interact with four carbonyl groups within the convergent channel, yielding a reduced interaction distance of approximately 6.5 Å, whereas the AA‐stacked COF accommodates Zn^2+^ through interaction with six carbonyl groups at a substantially larger average distance of about 10.5 Å (Figures S15 and S16). This spatial separation clearly exceeds the typical Zn–O coordination distance, indicating that the interaction is not a direct coordination [23, 24]. Instead, it arises from the cooperative long‐range electrostatic potential of multiple carbonyl groups, effectively providing outer‐sphere‐like stabilization. The computed adsorption energy reflects the overall multi‐dentate interaction of Zn^2^^+^ with multiple pore carbonyl groups, providing a meaningful physical measure of its association tendency within the COF environment rather than a conventional single‐ligand metal complexation energy. The corresponding calculated adsorption energies are summarized in Figure 3b, showing that TPDB‐AB exhibits a markedly stronger interaction with Zn^2+^ at 304.69 kJ mol^−1^ compared with 184.92 kJ mol^−1^ for TPDB‐AA. This difference arises from the staggered stacking‐induced pore contraction in TPDB‐AB, which optimizes the spatial arrangement of carbonyl groups and places them at more favorable coordination distances from the Zn^2+^ center, thereby enhancing orbital overlap and electrostatic interaction.

To assess the reliability of this approach for evaluating the interactions between Zn^2+^ and COF molecular skeleton, the adsorption energy between Zn^2+^ and a single water molecule was calculated under the same solvent conditions. The resulting value (−195 kJ mol^−1^) closely matches the theoretical dehydration energy of the first water molecule with Zn^2+^ (around −200 kJ mol^−1^), providing a quantitative reference for the energetic scale of Zn^2+^‐carbonyl coordination within in COFs channel. Although the calculated adsorption energy of Zn^2+^ in the AB‐stacked COF remains lower than the hydration energy of Zn^2+^ (∼2000 kJ mol^−1^), this discrepancy originates from the use of a limited number of COF layers in the computational model. In realistic multilayer COF membranes, additional carbonyl sites along the transport pathway can participate cooperatively in coordination, providing cumulative stabilization that is not fully captured in the bilayer approximation. These results indicate that the AB‐staggered topology intrinsically tailors the pore geometry and chemical environment, leading to stronger Zn^2+^ adsorption through optimized interaction distances. Such stacking‐induced structural confinement is therefore expected to partially compensate the energetic penalty associated with Zn^2+^ dehydration and reduce the overall solvation barrier during ion migration.

Based on this interaction mechanism, molecular dynamics (MD) simulations were further conducted to elucidate how different interlayer stacking modes of COF membranes regulate the transport kinetics and hydration structure of hydrated zinc ions at the molecular level. While MD simulations have inherent limitations in modeling water transport within confined nanopores, here this approach is used to examine the electrolyte structure of hydrated Zn^2^^+^ after passing through two‐layer COF frameworks [25, 26, 27, 28]. By applying identical simulation conditions, the approach allows comparison of AA‐ versus AB‐stacked pores, qualitatively analysis of COF topology‐dependent partial Zn^2^^+^ dehydration. A 2 m ZnSO_4_ electrolyte model was constructed as shown in Figure 3c,d. Two bilayer COF membranes with distinct AA and AB stacking configurations were placed at the center of the simulation box. The electrolyte was then driven across the membranes under an external electric field, and the transmembrane transport behavior and coordination environments of each species were quantitatively analyzed. The results show that the permeation rate of water molecules through the AB stacked COF membrane is lower than that through the AA stacked membrane (Figure 3e), whereas the transport rate of Zn^2+^ ions across the AB stacked membrane is markedly higher than that of the AA stacked counterpart (Figure 3f), indicating that the AB stacking architecture enables preferential Zn^2+^ transport while suppressing water migration. Coordination analysis of Zn^2+^ after transmembrane transport further reveals that the average hydration number of Zn^2+^ decreases more significantly after passing through the AB stacked COF membrane than through the AA stacked membrane, as shown in Figure 3g,h, demonstrating that the compact nanochannels formed by AB stacking promote stronger ion framework interactions and induce partial dehydration of Zn^2+^. This reduced hydration shell lowers the desolvation energy barrier and provides the thermodynamic and kinetic basis for accelerated Zn^2^^+^ transport through the AB‐stacked COF membrane in a low‐solvation state. To ensure the reproducibility and reliability of the MD simulations, two additional independent MD simulations were performed (Figures S17 and S18 and Video S1). Both simulations consistently show that, compared with the AA‐COF membrane, the ZnSO_4_ electrolyte passing through the AB‐COF membrane exhibits clear selective transport of Zn^2^^+^ ions and water molecules, along with a significant reduction in Zn^2^^+^ hydration.

For experimentally revealing the desolvation transport mechanism of Zn^2+^ in COF membranes with distinct stacking configurations, multiple spectroscopic techniques were employed to provide insights by studying the chemical environment of water molecules. X‐ray photoelectron spectroscopy (XPS) of Zn^2+^ confined in the two COF membranes shows that the Zn 2p spectra display single, unshifted peaks in both COF membranes (Figure 3i), indicating nearly identical chemical environments and excluding the presence of direct coordination interactions. This observation instead supports a transport scenario dominated by long‐range collective electrostatic potentials within the nanochannels. ^1^H NMR spectra of ZnSO_4_ electrolytes confined in the two COF membranes were further collected and compared with that of pure water to probe the local environment of water molecules (Figure 3j). The water proton resonance in the AB‐stacked COF membrane is closer to those of bulk water, implying a substantially reduced probability of Zn^2+^‐water coordination relative to the AA‐stacked membrane and the pristine electrolyte, which indirectly evidences the enhanced Zn^2+^ desolvation capability of the TPDB‐AB membrane. ATR‐FTIR and Raman spectroscopy were also applied to quantitatively analyze the water states within the two membranes (Figure 3k and Figure S19), with the fractions of water species at different adsorption strengths summarized in Figure 3l and Figure S20. Because ATR‐FTIR and Raman spectroscopy possess different vibrational sensitivities and selection rules, the absolute fractions obtained from the two methods may differ slightly. However, both analyses reveal the same overall trend. Compared with water confined in the AA‐stacked COF membrane and in the ZnSO_4_ solution, water within the AB‐stacked COF membrane exhibits a markedly higher proportion of free water species. This result supports the proposed topology‐dependent desolvation and ion‐transport mechanism and further confirms the stronger ability of the AB‐stacked COF membrane to induce Zn^2^^+^ desolvation during transmembrane transport.

**FIGURE 4:**
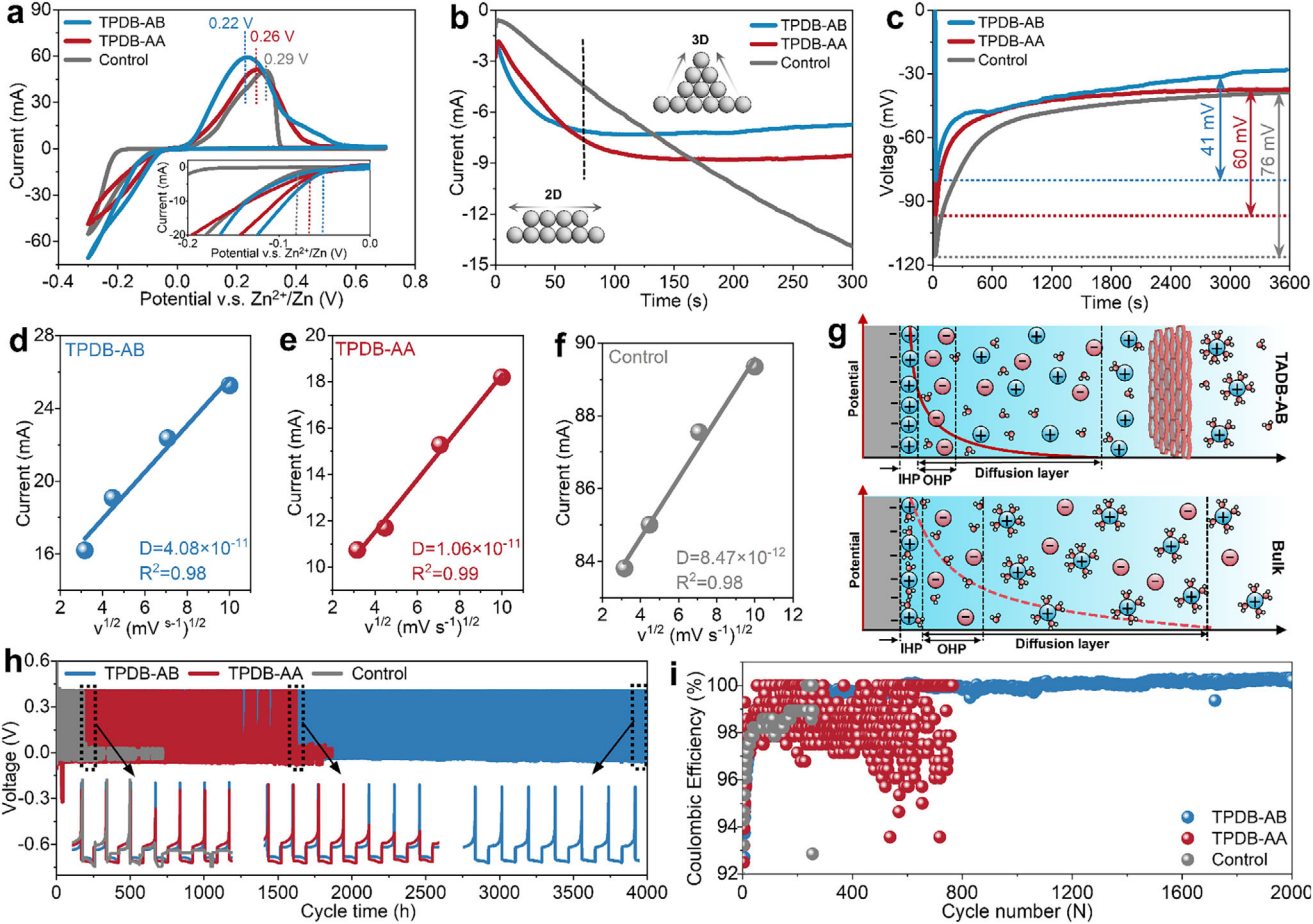
Electrochemical behavior of Zn^2+^ regulated by TPDB‐AA and TPDB‐AB membranes. (a) CV curves, (b) CA curves at a bias potential of −150 mV, and (c) CP curves at 1 mA cm^−2^ of Zn||Cu cells paired with TPDB‐AA and TPDB‐AB membranes. Peak current vs. square root of scan rate plots for Zn||Cu cells using (d) TPDB‐AB, (e) TPDB‐AA, and (f) control sample using glass fiber separators. (g) the image of bound water molecules in the inner Helmholtz layer. (h) The charge–discharge profiles of Zn||Cu cells (current density:10 mA cm^−2^ and areal capacity: 10 mAh cm^−2^) and (i) corresponding Coulombic efficiency with different membranes.

Building on the distinct ion transmembrane transport characteristics imparted by different COF stacking configurations, we further examined how such ion regulation influences the electrochemical deposition behavior of Zn^2+^ in order to clarify the electrochemical responses of the underlying ion transport mechanisms. Zn||Cu asymmetric cells were employed as a platform to probe the electrochemical behaviors of Zn^2+^ deposited on the clean copper substrate. Cyclic voltammetry (CV) measurements of Zn plating and stripping were first collected, as shown in Figure 4a. Zn^2+^ regulated by the TPDB‐AB membrane exhibits a substantially lower nucleation overpotential than that regulated by the TPDB‐AA membrane and the bulk ZnSO_4_ electrolyte, which can be attributed to the reduced desolvation penalty of partially dehydrated Zn^2+^ species reaching the electrode surface and thereby lowering the energy barrier for nucleation. Meanwhile, Zn oxidation under TPDB‐AB regulation occurs at a lower potential and displays a broader stripping peak, indicating a more gradual and spatially distributed stripping process that is consistent with a low‐solvation Zn^2+^ environment and weakened Zn‐H_2_O coordination during interfacial charge transfer [24, 29]. Chronoamperometry (CA) curves recorded at a bias potential of 150 mV further reveal that Zn deposition currents regulated by the TPDB‐AB membrane are smaller and reach a steady plateau more rapidly than those observed with the TPDB‐AA membrane and the bulk electrolyte, reflecting a faster establishment of stable nucleation sites and diffusion fields enabled by desolvated Zn^2+^ transport (Figure 4b and Figure S21). Chronopotentiometry (CP) measurements (Figure 4c) conducted at a current density of 1 mA cm^−2^ show trends consistent with the CV results and also demonstrate a markedly reduced nucleation overpotential under TPDB‐AB regulation, collectively confirming that the transmembrane desolvation and fast transport behavior of Zn^2+^ directly improve its electrochemical deposition kinetics and interfacial stability.

To further examine how COF membranes with different stacking configurations regulate Zn^2+^ transport at the electrode interface, CV measurements were performed at varying scan rates (Figure S22). By fitting the linear relationship between the peak current and the square root of the scan rate, the apparent diffusion coefficients of Zn^2+^ at the electrode surface were determined (Figure 4d–f). The Zn^2+^ diffusion coefficient induced by the TPDB‐AB membrane reaches 4.08 × 10^−11^ cm^2^ s^−1^, which is markedly higher than that obtained with the TPDB‐AA membrane at 1.06 × 10^−11^ cm^2^ s^−1^ and the bulk electrolyte at 8.47 × 10^−12^ cm^2^ s^−1^. In parallel, the electric double layer (EDL) capacitance at the electrode surface was likewise evaluated, revealing that in 2 m ZnSO_4_ electrolyte, the TPDB‐AB membrane induces an obviously lower EDL capacitance than both the TPDB‐AA membrane and the bulk electrolyte (Figure S23). From the perspective of classical EDL theory, a reduced capacitance reflects a more compact interfacial charge distribution and a shortened effective double‐layer thickness, which can be attributed to the partial desolvation of Zn^2+^ prior to reaching the electrode surface. Dehydration‐induced decrease in the effective dielectric constant and suppressed water polarization within the reconstructed EDL [30, 31]. The diminished hydration shell reduces the accumulation of strongly bound water molecules in the inner Helmholtz layer (Figure 4g). Therefore, it weakens interfacial polarization and suppresses water‐related parasitic reactions, thereby benefiting the electrochemical interfacial stability.

As a result, even under the same ZnSO_4_ electrolyte composition, the TPDB‐AB membrane significantly broadens the electrochemically stable voltage window, as evidenced by the linear sweep voltammetry shown in Figure S24. These results indicate that the TPDB‐AB membrane induces a compact and Zn^2+^‐dominated EDL structure characterized by reduced water molecule participation and accelerated interfacial charge transfer. The cycling stability of Zn plating and stripping on Cu substrates was further evaluated by cyclic charge–discharge test (Figure 4h and Figures S25 and S26), where cells employing the TPDB‐AB membrane exhibit stable voltage profiles over 2000 continuous cycles at a large current density of 10 mA cm^−2^ (areal capacity: 10 mAh cm^−2^). Notably, the corresponding Coulombic efficiency remains close to 99.7 ± 0.3% throughout 2000 cycles, indicating highly reversible Zn deposition and the effective suppression of parasitic reactions such as hydrogen evolution and corrosion even under deep electrochemical stripping and deposition conditions. Although slight fluctuations in Coulombic efficiency were observed during long‐term testing under ambient conditions due to environmental interference, the overall Coulombic efficiency remained highly stable throughout the cycling process without evidence of short‐circuit failure. In contrast, cells using the TPDB‐AA membrane exhibit more noticeable fluctuations in Coulombic efficiency during the early cycling stage compared with those using the TPDB‐AB separator, suggesting more pronounced parasitic reactions (Figure 4i). As the cycling proceeds, the TPDB‐AA‐based cells gradually become unstable and eventually undergo a typical soft short circuit at approximately 800 cycles (∼1600 h), whereas cells with the bulk electrolyte fail earlier at around 200 cycles (∼400 h) (Figure 4h). These results collectively demonstrate that the TPDB‐AB COF membrane establishes an efficient transmembrane transport pathway, where Zn^2+^ ions undergo partial desolvation during migration, enabling rapid and stable delivery to the electrode surface and thereby supporting the highly reversible Zn stripping and plating behavior. To the best of our knowledge, this unique electrolyte ion migration mechanism, characterized by membrane‐regulated ion distribution and interfacial migration at the electrode surface, has not previously been explored in aqueous battery systems.

### 2.3 Electrochemical Performance of TPDB‐AA and TPDB‐AB Membranes

**FIGURE 5:**
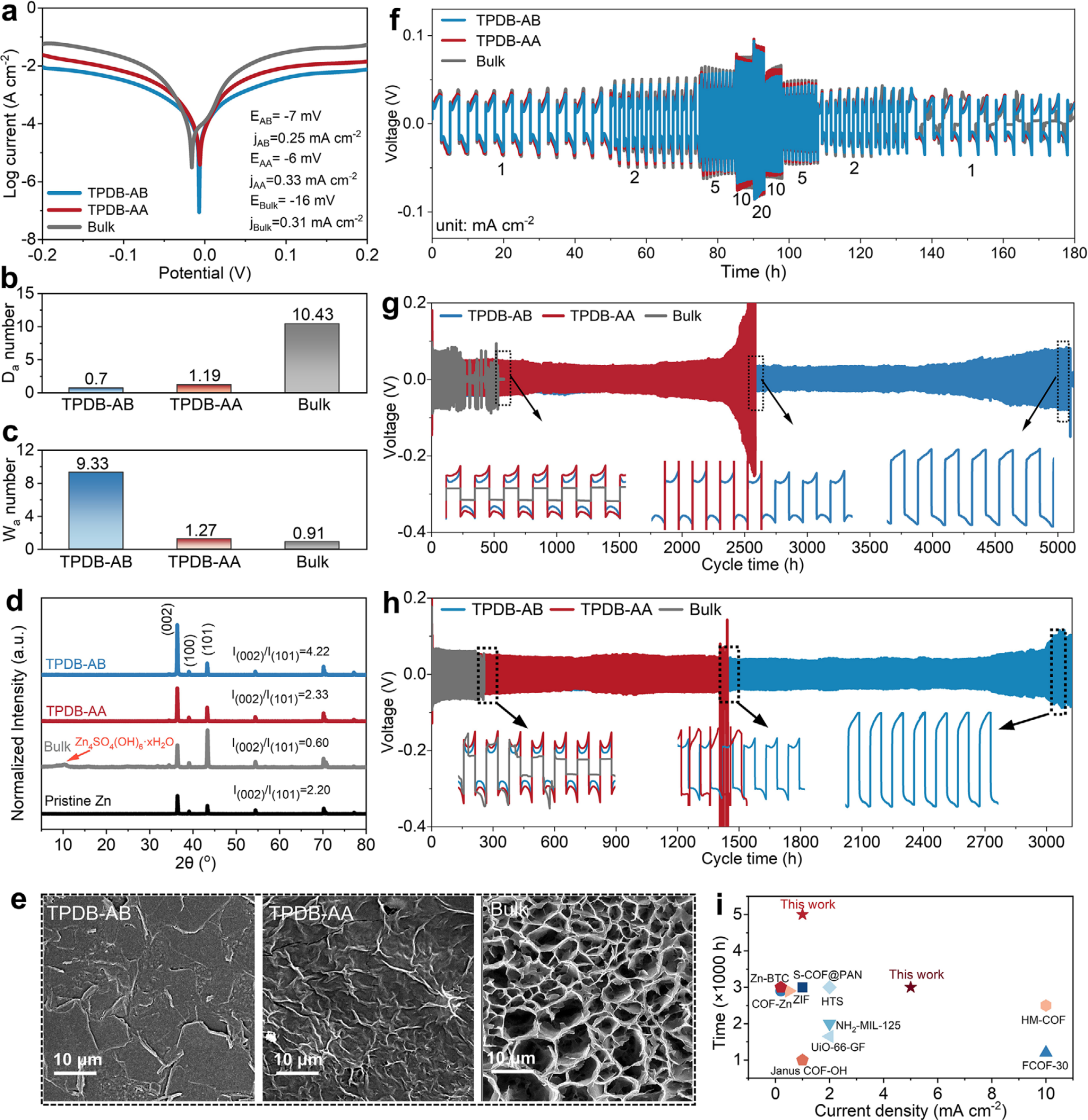
Cyclic performance of Zn electrode in 2 m ZnSO_4_ electrolyte regulated by COF membranes with different configurations. (a) Tafel plots, (b) *D_a_* numbers, and (c) *W_a_* numbers of Zn electrode regulated by different COF membranes. (d) XRD patterns and (e) SEM images of Zn electrode after 100th cycles. (f) Rate performance of Zn||Zn cells paired with different membranes. Charge–discharge cycling profiles of Zn||Zn cells with different COF membranes at a current density of (g) 1 mA cm^−2^ (areal capacity: 1 mAh cm^−2^) and (h) 5 mA cm^−2^ (areal capacity: 5 mAh cm^−2^). (i) Performance summary of the cycling lifetime of Zn||Zn cells using different separator materials at various current densities.

We evaluated the electrochemical performance of COF membranes with different configurations in multiple electrochemical cell systems. We first systematically investigated the cycling stability and underlying electrochemical mechanisms of symmetric Zn–Zn cells mediated by different COF membranes. Tafel polarization curves were collected for symmetric cells assembled with different COF membranes, as shown in Figure 5a, to elucidate the corrosion behavior and interfacial reaction kinetics of the Zn electrodes. The cell employing the TPDB‐AB membrane exhibits the lowest corrosion current density of 0.25 mA cm^−2^, which is significantly lower than that of cells using the TPDB‐AA membrane at 0.33 mA cm^−2^ and the bulk electrolyte at 0.31 mA cm^−2^. Meanwhile, the corrosion potential of the TPDB‐AB‐based cell shifts to a more positive value relative to the bulk electrolyte and TPDB‐AA counterparts. These results indicate that the TPDB‐AB membrane effectively suppresses spontaneous Zn corrosion and parasitic hydrogen evolution while reconstructing the Zn‐electrolyte interface toward a more stable electrochemical equilibrium. This behavior can be attributed to the regulated Zn^2+^ desolvation process within the ordered nanochannels of the TPDB‐AB membrane, which lowers the interfacial energy barrier for Zn^2^^+^ transfer and reduces the driving force for undesired side reactions.

To further clarify the interplay between ion transport and electrochemical reduction kinetics, the Damköhler (*Da*) number and Wagner (*Wa*) number were determined for cells with different COF membranes [32, 33, 34]. As shown in Figure 5b,c and Figure S27 and Table S2, the TPDB‐AB‐based cell exhibits a lower *Da* number and a higher *Wa* number than those using the TPDB‐AA membrane and the bulk electrolyte. A higher *Wa* number reflects faster Zn^2+^ transport, which promotes a more homogeneous ion concentration distribution at the electrode surface and suppresses local current density amplification. Concurrently, the reduced *Da* number indicates a pronounced decrease in heterogeneous nucleation sites, thereby mitigating stochastic Zn growth. From a kinetic perspective, the combination of a low *Da* number and a high *Wa* number suggests that Zn deposition is predominantly governed by ion diffusion in the electrolyte rather than being limited by interfacial electrochemical reduction. From a thermodynamic perspective, the moderated Zn^2+^ desolvation and reduced interfacial energy fluctuations favor a more uniform nucleation landscape, which is conducive to lateral and energetically preferred Zn growth. XRD patterns shown in Figure 5d and SEM images in Figure 5e further substantiate the distinct deposition mechanisms induced by different membranes. In COF membrane‐based cells, the Zn (002) plane becomes the dominant diffraction feature, indicating planar and horizontal Zn deposition on the electrode surface. In contrast, cells using the bulk electrolyte are dominated by the Zn (101) plane, which corresponds to vertically oriented and randomly distributed growth that readily evolves into dendrites. SEM observations clearly reveal that the TPDB‐AB membrane induces more uniform, compact, and smooth Zn deposits compared with both the bulk electrolyte and the TPDB‐AA membrane. This behavior is further confirmed by Zn deposition experiments on Cu substrates, as shown in Figures S28 and S29, demonstrating that membrane‐mediated Zn^2+^ desolvation enables effective regulation of the crystallographic orientation of Zn deposition and enables homogeneous lateral growth, thereby substantially enhancing the cycling stability of Zn electrodes in aqueous electrolyte.

We further evaluated the rate performance of symmetric cells assembled by different COF membranes. As shown in Figure 5f, cells assembled with the TPDB‐AB and TPDB‐AA membranes exhibit highly stable and symmetric polarization voltage profiles over a wide range of current densities, indicating favorable electrochemical reversibility and interfacial stability. In contrast, the cell employing the bulk electrolyte shows pronounced voltage fluctuations during cycling and ultimately suffers from severe short‐circuit failure. At a standard current density of 1 mA cm^−2^ (areal capacity: 1 mAh cm^−2^), the Zn||TPDB‐AB||Zn cell delivers stable plating/stripping more than 5000 h during prolonged cycling tests, which represents a substantial improvement compared with the Zn||TPDB‐AA||Zn cell with approximately 2500 h and the Zn||Bulk||Zn cell with only about 200 h, as shown in Figure 5g. In comparison, Zn||Zn symmetric cells assembled with pure cellulose and cellulose/POM composite membranes exhibited much shorter lifetimes of less than ∼800 and ∼1300 h, respectively (Figures S13 and S14). Once the current density was increased to 5 mA cm^−2^ (areal capacity: 5 mAh cm^−2^), the Zn||TPDB‐AB||Zn cell still maintains stable operation for over 3000 h, as shown in Figure 5h, further confirming its outstanding cycling robustness. We compared the performance of recently reported strategies to enhance the cycling stability of the zinc anode. Comparisons with porous framework‐based separators show that the AB‐COF membrane exhibits superior cycling lifetimes under similar testing conditions (Figure 5i, Table S3). Moreover, even compared with systems using electrolyte additives or interfacial engineering strategies, the AB‐COF membrane exhibits superior cycling stability (Table S4), underscoring the critical role of its AB‐staggered structural design in enabling enhanced electrochemical performance. In addition, the stripping and plating behavior under deep discharge conditions with a depth of discharge of 50% was investigated for cells with different separators. The Zn||TPDB‐AB||Zn cell exhibits markedly enhanced stability during deep stripping and plating cycling, as shown in Figure S30, whereas the Zn||TPDB‐AA||Zn cell fails after a limited number of cycles, and the Zn||Bulk||Zn cell is unable to sustain effective deep stripping and plating throughout the test. These results collectively demonstrate that the TPDB‐AB COF membrane significantly enhances the stability and reversibility of Zn electrodes in aqueous electrolytes by regulating ion transport and the interfacial environment. The structural stability of the COF membranes under electrochemical operation was also examined. As shown in Figures S31–S34, the COF membranes retain a uniform thickness morphology after repeated charge and discharge cycles. The COF crystals remain homogeneously distributed without noticeable detachment, while their chemical structure also remains stable during cycling. These results indicate the excellent mechanical robustness, chemical stability, and structural integrity of the membranes during prolonged battery operation.

**FIGURE 6:**
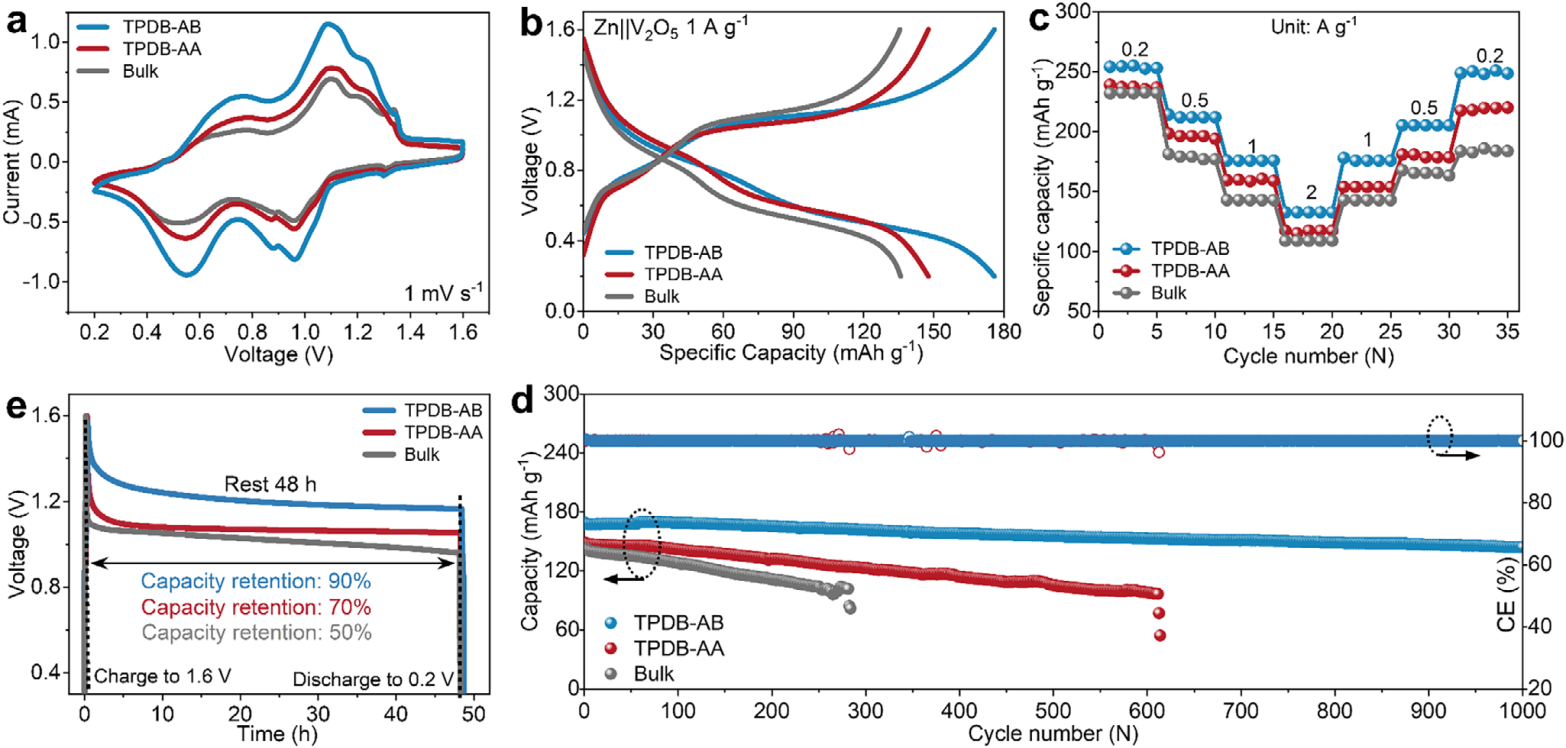
Full battery performance assembled by COF membranes with different configurations. (a) CV curves, (b) charge–discharge curves, (c) rate performance, (d) long‐term cycling discharge capacity and Coulombic efficiency with a current density of 1 A g^−1^, (e) self‐discharge profiles of Zn||V_2_O_5_ full batteries after 48 h rest with different COF membranes.

We further evaluated the electrochemical performance of COF membranes with different configurations in Zn‐V_2_O_5_ full battery systems. CV results in Figure 6a show that the Zn||TPDB‐AB||V_2_O_5_ batteries exhibit a substantially larger integrated CV area than the Zn||TPDB‐AA||V_2_O_5_ and Zn||Bulk||V_2_O_5_ batteries, indicating enhanced charge storage capability. Consistently, the corresponding galvanostatic charge and discharge profiles reveal higher discharge capacities and reduced polarization for the TPDB‐AB‐based battery (Figure 6b). It has been well recognized that the V_2_O_5_ cathode stores energy primarily through a reversible multistep Zn^2+^ intercalation and deintercalation process accompanied by lattice distortion, during which co‐intercalated water molecules can substantially disrupt lattice stability, thereby necessitating low‐solvation states of Zn^2+^ and their efficient transport kinetics. In this context, the ordered nanochannels of the TPDB‐AB membrane facilitate rapid Zn^2+^ conduction and promote partial desolvation prior to interfacial insertion, thereby lowering the energy barrier for Zn^2+^ diffusion into the V_2_O_5_ host and enabling more complete utilization of electrochemically active sites, which accounts for the increased capacity. The rate capability of full batteries with different COF membranes was further examined, as shown in Figure 6c. The Zn||TPDB‐AB||V_2_O_5_ battery delivers consistently higher capacities over a wide range of current densities and exhibits superior capacity recovery when the current density is returned to lower values, demonstrating fast electrochemical kinetics and good structural reversibility. This favorable rate performance originates from the synergistic effects of accelerated Zn^2^^+^ transport and regulated desolvation within the TPDB‐AB membrane, which together minimize concentration polarization and interfacial resistance under high‐rate conditions.

Long‐term cycling tests conducted at a large current density of 1 A g^−1^ show that the Zn||TPDB‐AB||V_2_O_5_ battery maintains a discharge capacity above 165 mAh g^−1^ after 1000 cycles, corresponding to more than 90% of the initial capacity, while the Coulombic efficiency remains close to 100% throughout the cycling process, as shown in Figure 6d. By contrast, the Zn||TPDB‐AA||V_2_O_5_ and Zn||Bulk||V_2_O_5_ batteries exhibit pronounced capacity decay during cycling and suffer from evident short‐circuit failure after approximately 610 and 290 cycles, respectively. This indicates that zinc ions partially desolvated by the TPDB‐AA membrane effectively mitigate capacity fading associated with lattice distortion in the V_2_O_5_ cathode. In addition, the self‐discharge behavior of full batteries with different COF membranes was evaluated by charging the cells to 1.6 V followed by a long‐term rest period. As shown in Figure 6e, the Zn||TPDB‐AB||V_2_O_5_ battery retains 90% of its initial capacity, whereas the Zn||TPDB‐AA||V_2_O_5_ and Zn||Bulk||V_2_O_5_ batteries show capacity retention values of 70% and 50%, respectively. These results indicate that the TPDB‐AB membrane effectively suppresses self‐discharge in aqueous batteries, which can be attributed to its ability to decrease Zn^2+^ desolvation and fast ion transport, thereby reducing parasitic reactions and mitigating spontaneous redox processes and ion redistribution that commonly drive self‐discharge in aqueous battery systems.

## 3 Conclusions

In summary, we draw inspiration from the efficient passive ion transport of biological membranes and translate key mechanistic features into a synthetic COF membrane system for aqueous zinc batteries. Biological ion channels achieve rapid and selective ion transport through a dehydration‐compensation mechanism, in which spatially arranged electron‐rich functional groups transiently coordinate permeating ions and fully offset the energetic penalty associated with hydration shell removal. Such an energy‐balanced transport mode represents an ideal yet rarely realized paradigm for aqueous electrochemical systems, where sluggish ion desolvation and severe interfacial side reactions often limit performance and stability. Although COF membranes are rigid crystalline structures without intrinsic dynamic gating, the analogy to biological ion channels provides a conceptual framework to understand confined‐ion dehydration and transport, and the observed behaviors in AB‐COF membranes are governed by partial desolvation and structural constraints rather than by dynamic structural changes. By systematically examining the structural and electrochemical behavior of *β*‐ketoenamine‐linked COF with different stacking configurations, we demonstrate that staggered AB‐stacked COF membranes can partially emulate this biological strategy. The convergent nanochannels formed by the staggered topology are enriched with carbonyl groups that act as electron‐rich sites, enabling compact, coordination‐like multisite interactions with zinc ions. These interactions dynamically stabilize partially dehydrated ions, reduce the effective desolvation barrier, and promote fast and homogeneous ion transport through the confined channels. As a result, the Zn^2+^ flux at the electrode interface is significantly homogenized, which suppresses dendritic growth and mitigates parasitic reactions such as hydrogen evolution. Electrochemical evaluations corroborate this mechanistic picture. AB‐stacked COF membranes enable zinc metal anodes to operate with ultra‐long cycling stability exceeding 5000 h in a conventional 2 m ZnSO_4_ electrolyte without additives. In Zn‐V_2_O_5_ full batteries, the same membranes deliver high reversible capacities with more than 90% capacity retention after 1000 cycles at a high current density of 1 A g^−1^, outperforming most recently reported separator systems. Although the staggered COF membranes presented herein cannot achieve the ideal ion dehydration transport characteristic of biological membranes with zero‐energy input, the demonstrated biomimetic transport behavior and its pronounced efficiency in aqueous batteries provide valuable theoretical insight. This work highlights how topological control and chemical environment engineering in COF membranes can be leveraged to regulate ion desolvation and interfacial processes, offering guiding principles for the rational design of COF‐based membranes and their application in electrochemical energy storage systems.

## Author Contributions

**Zihan Tan**: investigation, Writing – review and editing. **Zhixiang Dai**: investigation, writing – review and editing. **Shengyang Zhou**: conceptualization, methodology, software, investigation, validation, formal analysis, data curation, supervision, project administration, funding acquisition, resources, writing – original draft, writing – review and editing, visualization. **Jiaxin Liu**: investigation, formal analysis, writing – review and editing. **Zhong‐Ming Li**: investigation, project administration, supervision, writing – review and editing. **Hongli Yang**: investigation, writing – review and editing. **Xuan Li**: investigation, formal analysis, writing – original draft, writing – review and editing. **Yilin Zhang**: investigation, writing – review and editing. **Chao Xu**: investigation, writing – review and editing.

## Conflicts of Interest

The authors declare no conflicts of interest.

## Supporting information




**Supporting File 1**: advs76918‐sup‐0001‐SuppMat.docx.

**Supporting File 2**: advs76918‐sup‐0002‐VideoS1.mp4.

## Data Availability

The data that support the findings of this study are available from the corresponding author upon reasonable request.
