# Supplementary material for: A Covalent Organic Framework With Staggered Topology for Mimetic Transmembrane Transport of Hydrated Ions
Source: Adv Sci (Weinh). 2026 Aug 5;13(60):e76918. doi: 10.1002/advs.76918 (PMC13442310; doi:10.1002/advs.76918)
Supplement: Supplementary file 1 [file ADVS-13-e76918-s002.docx]

**Supporting Information**

**A Covalent Organic Framework with Staggered Topology for Mimetic Transmembrane Transport of Hydrated Ions**

*Xuan Li^1^, Jiaxin Liu^1^, Yilin Zhang^1^, Zhixiang Dai^1^, Zihan Tan^1^, Hongli Yang^1^, Chao Xu^2^, Shengyang Zhou ^1, *^, Zhong-Ming Li^1^*

^1^College of Materials Science and Engineering, College of Polymer Science and Engineering, State Key Laboratory of Advanced Polymer Materials, Sichuan University, Chengdu 610065, China

^2^Department of Materials Science and Engineering, The Ångström Laboratory, Uppsala University, Uppsala 752 37, Sweden

*^*^*E-mail: [shengyang.zhou@scu.edu.cn](mailto:shengyang.zhou@scu.edu.cn)

**Experimental Section**

**Materials.** Zinc sulfate heptahydrate (ZnSO_4_·7H_2_O, AR, Aladdin), zinc foil (50 μm, Olegeeino), cooper foil (50 μm, Olegeeino), glass fiber (Olegeeino), cellulose (Cellubio), 2,4,6-triformylphloroglucinol (TP, Aladdin), benzene-1,4-diamine (DB, Aladdin), acetic acid (AC, Aladdin), phosphomolybdic acid (H_3_[Mo_12_PO_40_]_12_ ∙ H_2_O, Aladdin) was used without any treatment. V_2_O_5_ cathode were provided by Future (Jilin) Materials Tech. Co. Ltd. The water used in the study was ultra-pure (18.2 MΩ · cm), produced by an ultra-pure water system (Shanghai Lichen Instrument Technology Co., Ltd.).

**Figure S1.** Synthesis of TPDB-AB COF.

**Synthesis of TPDB-AB COF**. Add 129 mg (0.6 mmol) of 2,4,6-Trimethoxy-1,3,5-benzenetricarbaldehyde (TP) to a 20 mL glass vial. Subsequently, add 2 mL of 6 M acetic acid (AC) aqueous solution and 2 mL polyoxometalate solution (2 mg mL^-1^) (phosphomolybdic acid with the formula H_3_[Mo_12_PO_40_]∙_12_H_2_O). Stir the mixture magnetically at 400 rpm for 30 minutes at room temperature to dissolve the powder completely and ensure thorough mixing. Dissolve 98 mg (0.9 mmol) of benzene-1,4-diamine (DB) in 4 mL of ultrapure water to obtain a clear solution. Under continuous stirring, slowly add the amine solution to the reaction flask using a dropper, controlling the addition rate to approximately 1 mL min^-1^ to avoid localized overconcentration. After completion of the addition, seal the reaction vial and continue stirring at room temperature for 72 hours. After the reaction, the solid product was collected by centrifugation (8000 rpm, 10 min) and washed three times with acetone to remove unreacted monomer and catalyst. The washed solid precipitate was redispersed in ultrapure water, sonicated for 10 min to obtain a homogeneous COF slurry for subsequent membranes preparation (Figure S1).

**Figure S2.** Synthesis of TPDB-AA COF.

**Synthesis of TPDB-AA COF.** Add 129 mg (0.6 mmol) of TP to a 20 mL glass vial. Subsequently, add 4 mL of 6 M AC solution. Stir the mixture magnetically at 400 rpm for 30 minutes at room temperature to dissolve the powder completely and ensure thorough mixing. Dissolve 98 mg (0.9 mmol) of benzene-1,4-diamine (DB) in 4 mL of ultrapure water to obtain a clear solution. Under continuous stirring, slowly add the amine solution to the reaction flask using a dropper, controlling the addition rate to approximately 1 mL min^-1^ to avoid localized overconcentration. After completion of the addition, seal the reaction vial and continue stirring at room temperature for 72 hours. After the reaction, the solid product was collected by centrifugation (8000 rpm, 10 min) and washed three times with acetone to remove unreacted monomer and catalyst. The washed solid precipitate was redispersed in ultrapure water, sonicated for 10 min to obtain a homogeneous COF slurry for subsequent membranes preparation (Figure S2).

**Schematic S1**. The detailed COF membranes preparation procedures.

**Preparation of membrane**s. Add 5 mL of the pre-prepared TPDB-AB or TPDB-AA COF slurry (50 mg mL⁻¹) to 40 mL of cellulose aqueous solution (2.5 mg mL⁻¹) and sonicate the mixture at room temperature for 10 minutes to achieve a uniform dispersion of COF particles within the cellulose. To ensure membrane homogeneity, the mixed suspension was poured onto a vacuum filtration apparatus in three portions: first 20 mL, then another 20 mL, and finally 10 mL. Each portion was vacuum-filtered under -0.08 MPa until no visible liquid remained on the surface, forming a homogeneous wet membrane. The wet membrane on the filter was carefully transferred between two steel plates and dried overnight (12 h) in a 60 °C vacuum oven to remove moisture completely. The dried membrane was peeled from the filter and cut into 16 mm diameter discs using a circular punch. A detailed schematic of the preparation procedure is provided in Schematic S1.

**Materials Characterization.** The X-ray diffraction (XRD) profiles were collected by an X-ray diffractometer with Cu K_α_ radiation (DX-2700B, HAOYUAN Instrument). Transmission electron microscopy (TEM) images were acquired on FEI Tecnai G2 F20 S-TWIN. The scanning electron microscopy (SEM) images were collected by a Nova NanoSEM (450, FEI). A nitrogen adsorption-desorption analyzer (Ankersmid Belsorp-Max) was used to investigate the distribution of the separator pore size and diameter. The mechanical and wettability properties of separators were tested by a universal test machine (INSTRON 5967 Universal Testing Systems) and a contact angle measurement device (Kruss DSA30S), respectively. Fourier transform infrared spectroscopy (FTIR) was performed by using a Thermo Scientific Nicolet 6700. H magnetic resonance imaging (NMR spectroscopy) was performed on Bruker AV II-400 MHz. The Raman mapping and point spectrum were obtained by a Laser micro-Raman imaging spectrometer (DXIS, USA) with the wavelength of 532 nm. The X-ray photoelectron spectra (XPS) was recorded on Axis ultra DLD. Small angle X-ray scattering (SAXS) patterns were obtained by using a scatter-meter (Xeuss 3.0, France).

**Electrochemical Characterization.** The commercial Zn foil (50 μm) and Cu foil (50 μm) were cut into discs with a diameter of 12 mm. Zn||Zn, Zn||Cu cells were assembled using CR2032 coin-type cells by adding 100 μL 2 M ZnSO_4_ electrolyte at room temperature. Zn||V_2_O_5_ full batteries were assembled using CR2032 coin-type cells by adding 100 μL 2 M ZnSO_4_ electrolyte at room temperature (cathode mass loading: 1 mg cm^-2^). The voltage window for Zn||V_2_O_5_ full battery cycling test was set from 0.2 V to 1.6 V. Electrochemical measurements, including cyclic voltammetry (CV), chronoamperometry (CA), electrochemical impedance spectroscopy (EIS), Tafel plots, and chronopotentiometry (CP), were conducted using an electrochemical workstation (CHI660E, Chenhua). EIS tests were conducted over a frequency range of 0.05 to 10^5^ Hz. Tafel tests were performed between -0.2 V and 0.2 V under 1 mV s^-1^$.$ CV tests for Zn||Cu half cells were carried out between -0.2 V and 0.5 V at 1 mV s^-1^. The battery tests were implemented using a Neware battery system (CT-4008T).

**Modeling of SAXS data.** A generalized empirical two-region Guinier-Porod model (proposed by Hammouda^1^) with Guinier and Porod regions was used to fit the SAXS data. It describes the form factor for nonspherical objects with the following equation:

$$I\left( q \right)=\left\{ \begin{aligned} \frac{G}{q^{s}}\mathrm{ex}p \left( \frac{-q^{2}R_{g}^{2}}{3-s} \right)I\left( q \right)=\frac{G}{q^{s}}\exp(\frac{-q^{2}R_{g}^{2}}{3-s}) , q\leq q1 \\ I\left( q \right)=\frac{D}{q^{\alpha}} , q\geq q1 \end{aligned} \right.$$

where *q* is the scattering vector, *I(q)* is the scattering intensity, *s* is the “dimensionality” parameters, and *Rg* is the characteristic length for the size of the scattering object, *α* is the Porod exponent, and *G* and *D* are the Guinier and Porod scale factors, respectively.

With the requirement for the constraint of continuity of the intensity function, its double derivative yields a transition region at *q_1_* and the following relationships are obtained, equation:

$\left\{ \begin{aligned} D=Gexp\left( \frac{-q_{1}^{2}R_{g}^{2}}{3-s} \right)q_{1}^{\left( \alpha-s \right)} \\ q_{1}=\frac{1}{R_{g}}\left[ \frac{\left( \alpha-s \right)\left( 3-s \right)\left( \alpha-s \right)\left( 3-s \right)}{2} \right]^{1/2} \end{aligned} \right.$

**Ionic Conductivity Measurements.** The ionic conductivities (*σ*) of the separators were measured using two stainless-steel electrodes. The *σ* can be calculated through the following equation^2^:

$$\sigma=\frac{L}{RS}$$

where *L* is the thickness of the separator, 𝑅 is the resistance based on the EIS test, and *S* is the contact area between the separator and stainless-steel electrode.

**Zn^2+^ Transfer Number Measurements.** Zn^2+^ transfer numbers (*t_Zn_^2+^*) of different separators were measured by assembling Zn||Zn symmetric cells based on the following calculation equation^2^:

$$t_{{Zn}^{2+}}=\frac{I_{S}\left( \Delta V-I_{0}R_{0} \right)}{I_{0}\left( \Delta V-I_{S}R_{S} \right)}$$

where *∆𝑉* is the constant polarization voltage (50 mV), *I_0_* and *I_S_* are the initial current and the steady-state current based on CA test, respectively. *R_0_* and *R_s_* are the initial resistance and the stable resistance based on EIS test, respectively.

**Desolvation Activation Energy Measurements.** The desolvation activation energy (*E_a_*) of hydrated Zn^2+^ was measured using Zn||Zn cells at different temperatures. The *E_a_* can be calculated through the following equation^3^:

$$\frac{1}{R_{ct}}=Aexp(\frac{-E_{a}}{RT})$$

where *R_ct_* is the resistance obtained from the EIS spectra, *R* is the gas constant, and *T* is the thermodynamic temperature.

**Measurement of Zn^2+^ Diffusion Coefficient.** The diffusion coefficient of Zn^2+^ (*D*) was measured using Zn||Cu cells. Under different scan rates, CV curves are scanned in the same voltage range. The *D* can be calculated through the following equation^4^:

$$I_{p}=0.4463nFAC_{x}V^{1/2}\sqrt{\frac{nFD_{X}}{RT}}$$

where *I_p_* is the peak current, *n* is the number of electrons gained and lost in the electrode reaction, *F* is the Faraday constant, *A* is the contact area between the electrode plate and electrode solution, *C_x_* is the concentration of the Zn^2+^, *V* is the scanning rate.

**Measurement of *D_a_* and *W_a_* number**. *Damköhler* number (*D_a_*) can be obtained by the following equation^5^:

$$D_{a}=\frac{i_{0}}{i_{l}}$$

*i_0_* is the exchange current at the electrode which is obtained from GCD curves, *i_l_* is the diffusion-limited current in the electrolyte which is calculated from:

$$i_{l}=\frac{4FCD_{+}}{L}$$

*F* is the Faraday constant, *C* is the salt concentration in the electrolyte solution, *D_+_* is the cation diffusion coefficient, and *L* is the space between physically separated electrodes.

*Wagner* number (*W_a_*) can be obtained by the following equation^6^:

$$W_{a}=\frac{R_{ct}}{R_{Ω}}$$

*R_ct_* is the charge transfer resistance from the EIS spectra and *R_Ω_* electrolyte resistance from the EIS spectra.

**Measurement of EDL capacitance**. The capacitance (*C*) is decided to the linear relationship between capacitive current (*i_c_*) and scan rate (*v*), which can be achieved from the slope of the current-voltage curves. Thus, the *C* can be calculated by the following equation^7^:

$$C=\frac{i_{c}}{v}$$

where *i_c_=(i_0v+_-i_0v-_)/2*, meaning the half value of current difference during positive and negative scans at 0 V.

**Molecular dynamic (MD) simulations.** Zn^2+^ and H_2_O molecules passing through bilayer TPDB-AB COF and TPDB-AA COF. under an electric field with strength of 0.5 V Å^-1^ was simulated by molecular dynamic (MD) simulations. The sandwich model was built utilizing “build layer” tool in Materials Studio (MS) 2023. The solution was comprised of 100 ZnSO_4_ and 2775 H_2_O molecules. The dimensional sizes of TPDB-AB COF were 44.05 Å×39.20 Å in *xoy* plane, and 46.23 Å×40.04 Å for TPDB-AA COF. All MD simulations were carried out by Forcite module with COMPASS Ⅲ force field^8^ in MS 2023. Van der Waals and Coulomb interactions were respectively considered by atom based and Ewald methods with a cut-off value of 12.5 Å. Equations of motion were integrated with a time step of 1 fs. After energy minimization, the system was subjected to MD simulation for 500 ps in the NVT (T = 298.0 K) ensemble using the Nose thermostat. Detailed computational setting parameters are listed below:

**Table S1**. Detailed MD simulation parameters used in this work.

| **Setup** | **Parameters** |
| --- | --- |
| Thermostat | Nose thermostat |
| Cutoff | 12.5 Å |
| Time-step | 1 fs |
| Constraint algorithms | NVT |
| Electrostatic summation method | Atom based |
| vdW summation method | Atom based |
| Partial charge | Force field assigned |
| Energy | 1.0*10^-4^ kcal mol^-1^ |
| Force | 0.005 kcal mol^-1^Å^-1^ |
| Displacement | 5.0*10^-5^ Å^-1^ |
| Initial conditions | The solution was comprised of 100 ZnSO_4_ and 2775 H_2_O molecules. The dimensional sizes of TPDB-AB were 44.05 Å×39.20 Å in *xoy* plane, and 46.23 Å×40.04 Å for TPDB-AA. |

**Adsorption energy calculation.** Adsorption energies between ions and COF skeleton were calculated using density functional theory (DFT) with ORCA software. Simplified model complexes were fully optimized at the PBE0-D3/Def2-SVP level, including Grimme’s D3 dispersion correction and implicit solvation effects modeled via the CPCM approach with water as the solvent, while no symmetry constraints were applied and the default ORCA convergence criteria, which provide sufficiently high precision, were used. Single-point energies were subsequently computed at the ωB97M-V/Def2-TZVP level to obtain high-accuracy electronic energies, with effective core potentials (ECPs) applied to zinc atoms. As the calculations were performed for solvated isolated systems, no plane-wave basis set, cutoff energy, or periodic boundary conditions were employed. Adsorption energies (ΔE) were evaluated according to:

where $E_{\text{Zn-COF}}$is the total energy of the complex in solution, $E_{\text{Zn}}^{\text{solvated}}$is the energy of the solvated Zn^2+^, and $E_{\text{COF}}^{\text{solvated}}$is the energy of the COF fragment in the same solvent environment. It should be noted that this calculation considers the interaction between a single Zn²⁺ ion and multiple carbonyl groups, and that the spatial arrangement of these carbonyl groups differs between the AA-COF and AB-COF channels, which contributes to differences in the computed adsorption energies. Negative ΔE values indicate energetically favorable coordination, and basis set superposition error (BSSE) corrections were applied for selected systems to verify the reliability of the results, while simplified COF fragments were used to retain the key chemical environment.

**Supplementary Results**

**Figure S3.** Photos of (a) TPDB-AB, (b) TPDB-AA separators.

**Figure S4.** Side-view scanning electron microscopy (SEM) images of separators.

**Figure S5.** Energy-dispersive X-ray spectroscopy (EDS) mapping images of TPDB-AB separator, showing the uniform dispersion of TPDB-AB nanoparticles in the separator.

**Figure S6.** Energy-dispersive X-ray spectroscopy (EDS) mapping images of TPDB-AA separator, showing the uniform dispersion of TPDB-AA nanoparticles in the separator.

**Figure S7.** Nitrogen sorption isotherms and the corresponding pore size distributions of TPDB-AB COF.

**Figure S8.** Nitrogen sorption isotherms and the corresponding pore size distributions of TPDB-AA COF.

**Figure S9**. Contact angles test of separators with 2 M ZnSO_4_ electrolyte (a) TPDB-AB (b) TPDB-AA, suggesting their good wettability.

**Figure S10.** Nyquist plots of Zn||Zn symmetrical cell with (a) TPDB-AB, (b) TPDB-AA, (c) glass fiber (GF) separators at different temperatures.

**Figure S11.** Nyquist plots of different membranes and bulk 2M ZnSO_4_.

**Fig S12.** Chronoamperometry (CA) curves and ion transport number of Zn||Zn cells with (a) TPDB-AB, (b) TPDB-AA, (c) glass fiber (GF) separators, respectively. The bias voltage was set at 50 mV and the corresponding Nyquist plots were collected at the initial and steady states.

**Figure S13**. Preparation, structure, and electrochemical characterization of cellulose/POMs composite membranes. (**a**) Photo, (**b**) SEM image, (**c**) Nyquist plot, (**d**) Arrhenius activation energy of pure cellulose membrane, (**e**) Charge-discharge cycling profiles of Zn||Zn cells with cellulose/POMs composite membranes.

**Figure S14**. Preparation, structure, and electrochemical characterization of pure cellulose membranes. (**a**) Photo, (**b**) SEM image, (**c**) Nyquist plot, (**d**) Arrhenius activation energy of cellulose/POMs composite membrane, (**e**) Charge-discharge cycling profiles of Zn||Zn cells with pure cellulose membranes.

**Figure S15.** The coordination distance between Zn^2+^ and carbonyl groups in TPDB-AB COF.

**Figure S16.** The coordination distance between Zn^2+^ and carbonyl groups in TPDB-AA COF.

**Figure S17**. The second-time independently repeated molecular dynamics simulation of aqueous ZnSO_4_ electrolyte transport through the two COF membranes, including representative snapshots of 2 M ZnSO_4_ transport through two-layered TPDB-AB and TPDB-AA frameworks. Time-dependent transport numbers of H_2_O molecules and Zn^2+^ ions after passing the two COF membranes. Simulated radial distribution functions and coordination numbers of Zn^2+^ after passing through the TPDB-AA and TPDB-AB membranes.

**Figure S18**. The third-time independently repeated molecular dynamics simulation of aqueous ZnSO_4_ electrolyte transport through the two COF membranes, including representative snapshots of 2 M ZnSO_4_ transport through two-layered TPDB-AB and TPDB-AA frameworks. Time-dependent transport numbers of H_2_O molecules and Zn^2+^ ions after passing the two COF membranes. Simulated radial distribution functions and coordination numbers of Zn^2+^ after passing through the TPDB-AA and TPDB-AB membranes.

**Figure S19.** Raman spectra fitting of different COFs with 2 M ZnSO_4_ electrolyte (a) TPDB-AB COF, (b) TPDB-AA COF. (c) pure ZnSO_4_ electrolyte, (d) pure H_2_O.

**Figure S20.** The corresponding proportions of free water and bound water of ATR-FTIR of different COF with 2 M ZnSO_4_ electrolyte.

**Figure S21.** Zn||Cu asymmetric cell CA curves at a bias potential of 150 mV.

**Figure S22.** Cyclic voltammetry (CV) curves of Zn||Cu cells with (a) TPDB-AB, (b) TPDB-AA, (c) glass fiber (GF) separators, respectively, at various scanning rates.

**Figure S23**. EDL measurements for Zn foils in (a) TPDB-AB, (b) TPDB-AA and (c) glass fiber (GF) separators.

**Figure S24.** LSV curves of Zn||Zn symmetry cell at 1 mV S^-1^.

**Figure S25.** The charge-discharge curves of Zn||Cu asymmetric cells with different separators (current density:10 mA cm^-2^ and areal capacity: 10 mAh cm^-2^).

**Figure S26.** The initial and final charge-discharge curves of Zn||Cu asymmetric cells with (a) TPDB-AB, (b) TPDB-AA, (c) glass fiber (GF) separators at a current density of 0.5 mA cm^-2^.

**Figure S27**. Nyquist plots of Zn||Zn symmetrical cell with different separators and corresponding equivalent circuit fitting (inset).

**Table S2.** Fitting impedance parameters.

**Figure S28.** X-ray diffractometer (XRD) patterns of Cu foil after the first deposition cycle of Zn||Cu cells with TPDB-AB, TPDB-AA, and glass fiber (GF) separators, respectively (1 mA cm^-2^ and 1 mAh cm^-2^).

**Figure S29.** Scanning electron microscopy (SEM) of the deposition Zn images on Cu foil surface after the first deposition cycle of Zn||Cu cells (1 mA cm^-2^ and 1 mAh cm^-2^) with (a) TPDB-AB, (b) TPDB-AA, (c) glass fiber (GF).

**Figure S30.** Cycling performance of Zn||Zn symmetry cells with a deep Zn stripping (DOD=50%) under 1 mA cm^-2^.

**Figure S31.** Side-view scanning electron microscopy (SEM) of (a) TPDB-AB, (b) TPDB-AA separators after cycle at 1 mA cm^-2^.

**Figure S32.** Surface-view scanning electron microscopy (SEM) and corresponding energy X-ray spectroscopy (EDS) mapping images of TPDB-AB separator after cycle at 1 mA cm^-2^.

**Figure S33.** Surface-view scanning electron microscopy (SEM) and corresponding energy X-ray spectroscopy (EDS) mapping images of TPDB-AA separator after cycle at 1 mA cm^-2^.

**Figure S34**. FTIR spectrum of two COF membranes before and after cycling test.

**Table S3.** The performance comparison between TPDB-AB COF membrane and other reported porous framework materials separators for aqueous zinc-ion battery.

| **Materials** | **Design Principles** | **Zn\|\|Zn Cycle life / Current density** | | **Reference** |
| --- | --- | --- | --- | --- |
| S-COF@PAN | Constructing SO_3_H hydrophilic groups | | 3000 h  (1 mA cm^-2^) | *Adv. Mater.*  **2026**, e23580. |
| COF-Zn | Zn²⁺-substituted COF-SO_3_Zn_0.5_ | | 2900 h  (0.2 mA cm^-2^) | *Angew. Chem. Int. Ed.* **2025**, 64, e202423118. |
| FCOF-30 | Defect engineering constructs polarity sites and channels | | 1200 h  (10 mA cm^-2^) | *J. Am. Chem. Soc.*  **2025,** 147, 36626-36641. |
| NH_2_-MIL-125 | Charged channel electrostatic screening | | 2000 h  (2 mA cm^-2^) | *Adv. Energy Mater.*  **2025**, 15, e04201. |
| HTS | MOF in situ modification constructs zinc-affinity interfaces | | 3000 h  (2 mA cm^-2^) | *Nat. Commun.*  **2025**, 16, 259. |
| UiO-66-GF | MOF-limited-region-induced deposition | | 1650 h  (2 mA cm^-2^) | *Nano-Micro Letters*  **2022**, 14.1: 218. |
| ZIF | Melt quenching constructs an amorphous network | | 2900 h  (0.5 mA cm^-2^) | *Adva. Mater.*  **2025**, 37, 2413167. |
| HM-COF | Hollow COF microﬁbers | | 2500 h  (10 mA cm^-2^) | *Adva. Mater.*  **2026**, 38, e17197. |
| Janus COF-OH | Asymmetric interface engineering design | | 1000 h  (1 mA cm^-2^) | *Adv. Func. Mater.*  **2025***, e28150.* |
| Zn-BTC | Limited-domain channel reconstruction electrolyte | | 3000 h  (0.2 mA cm^-2^) | *Adva. Mater.*  **2020**, 32, 2004240. |
| TPDB-AB | Regulation of stacking patterns, carbonyl arrangement | | 5000 h  (1 mA cm^-2^) | **This work** |
| TPDB-AB | Regulation of stacking patterns, carbonyl arrangement | | 3000 h  (5 mA cm^-2^) | **This work** |

**Table S4.** The performance comparison between TPDB-AB membrane and other reported regulation approaches for aqueous zinc-ion battery.

| **Materials** | **Design Principles** | **Zn\|\|Zn Cycle life**  **/Current density** | **Reference** |
| --- | --- | --- | --- |
| TiBT_x_-31@Zn | Interfacial engineering | 2000 h  (1 mA cm^-2^) | *Angew. Chem. Int. Ed.* **2025**, 137, e202507504. |
| 5% TEP | Electrolyte regulation | 2704 h  (5 mA cm^-2^) | *Nat. Commun.*  **2026**, 17, 1690. |
| IL-Br | Gel electrolyte | 2500 h  (0.5 mA cm^-2^) | *J. Am. Chem. Soc.*  **2025**, 147, 46632-46641. |
| DEDBA-MCHD-Zn | Interfacial engineering | 1050 h  (5 mA cm^-2^) | *Adv. Mater.*  **2026**, e21198. |
| PE | Electrolyte additive | 700 h  (10 mA cm^-2^) | *Nat. Commun.*  **2025**, 16, 3142. |
| HE-50mM | Electrolyte regulation | 2000 h  (3 mA cm^-2^) | *Angew. Chem. Int. Ed.* **2025**, 137, e202422395. |
| BIL@Zn | Interfacial engineering | 2500 h  (1 mA cm^-2^) | *Matter*  **2025**, 8.11. |
| BE+SL | Electrolyte additive | 2800 h  (1 mA cm^-2^) | *Ener. Environ. Sci.*  **2026**, 19, 1385-1392 |
| ZSC 0.4 | Electrolyte additive | 2300 h  (1 mA cm^-2^) | *Adv. Energy Mater.*  **2026**, e06191. |
| Zn@Sc | Interfacial engineering | 2800 h  (1 mA cm^-2^) | *Adv. Mater.*  **2026**, e18003. |
| CZHE | Electrolyte regulation | 1200 h  (1 mA cm^-2^) | *Nat. Commun.*  **2025**, 16, 11069. |
| PS | Interfacial engineering | 1500 h  (5 mA cm^-2^) | *Ener. Environ. Sci.*  **2025**, 18, 9600-9610. |
| 2 M ZS+4 mM HDO | Electrolyte additive | 2600 h  (1 mA cm^-2^) | *Angew. Chem. Int. Ed.* **2025**, 137, e202518262. |
| BR-Zn | Interfacial engineering | 1900 h  (5 mA cm^-2^) | *Angew. Chem. Int. Ed.* **2023**, 135, e202308017. |
| TPDB-AB | Regulation of stacking patterns | 5000 h  (1 mA cm^-2^) | **This work** |
| TPDB-AB | Regulation of stacking patterns, | 3000 h  (5 mA cm^-2^) | **This work** |

**References**

1.	Hammouda, B., A new Guinier–Porod model. *Applied Crystallography* **2010,** *43* (4), 716-719.

2.	Yang, S.; Zhang, Y.; Zhang, Y.; Deng, J.; Chen, N.; Xie, S.; Ma, Y.; Wang, Z., Designing anti‐swelling nanocellulose separators with stable and fast ion transport channels for efficient aqueous zinc‐ion batteries. *Advanced Functional Materials* **2023,** *33* (42), 2304280.

3.	Zheng, Z.; Guo, S.; Yan, M.; Luo, Y.; Cao, F., A functional janus Ag nanowires/bacterial cellulose separator for high‐performance dendrite‐free zinc anode under harsh conditions. *Advanced Materials* **2023,** *35* (47), 2304667.

4.	Leftheriotis, G.; Papaefthimiou, S.; Yianoulis, P., Dependence of the estimated diffusion coefficient of LixWO3 films on the scan rate of cyclic voltammetry experiments. *Solid State Ionics* **2007,** *178* (3-4), 259-263.

5.	Jin, S.; Yin, J.; Gao, X.; Sharma, A.; Chen, P.; Hong, S.; Zhao, Q.; Zheng, J.; Deng, Y.; Joo, Y. L., Production of fast-charge Zn-based aqueous batteries via interfacial adsorption of ion-oligomer complexes. *Nature communications* **2022,** *13* (1), 2283.

6.	Kim, M.; Lee, J.; Kim, Y.; Park, Y.; Kim, H.; Choi, J. W., Surface overpotential as a key metric for the discharge–charge reversibility of aqueous zinc-ion batteries. *Journal of the American Chemical Society* **2023,** *145* (29), 15776-15787.

7.	Hu, Z.; Song, Z.; Huang, Z.; Tao, S.; Song, B.; Cao, Z.; Hu, X.; Wu, J.; Li, F.; Deng, W., Reconstructing hydrogen bond network enables high voltage aqueous zinc‐ion supercapacitors. *Angewandte Chemie* **2023,** *135* (38), e202309601.

8.	Akkermans, R. L.; Spenley, N. A.; Robertson, S. H., COMPASS III: Automated fitting workflows and extension to ionic liquids. *Molecular Simulation* **2021,** *47* (7), 540-551.
